# ADH IB Expression, but Not ADH III, Is Decreased in Human Lung Cancer

**DOI:** 10.1371/journal.pone.0052995

**Published:** 2012-12-28

**Authors:** Sarah C. Mutka, Lucia H. Green, Evie L. Verderber, Jane P. Richards, Doug L. Looker, Elizabeth A. Chlipala, Gary J. Rosenthal

**Affiliations:** 1 N30 Pharmaceuticals, Inc., Boulder, Colorado, United States of America; 2 Premier Laboratory, LLC, Longmont, Colorado, United States of America; University of Saarland Medical School, Germany

## Abstract

Endogenous S-nitrosothiols, including S-nitrosoglutathione (GSNO), mediate nitric oxide (NO)-based signaling, inflammatory responses, and smooth muscle function. Reduced GSNO levels have been implicated in several respiratory diseases, and inhibition of GSNO reductase, (GSNOR) the primary enzyme that metabolizes GSNO, represents a novel approach to treating inflammatory lung diseases. Recently, an association between decreased GSNOR expression and human lung cancer risk was proposed in part based on immunohistochemical staining using a polyclonal GSNOR antibody. GSNOR is an isozyme of the alcohol dehydrogenase (ADH) family, and we demonstrate that the antibody used in those studies cross reacts substantially with other ADH proteins and may not be an appropriate reagent. We evaluated human lung cancer tissue arrays using monoclonal antibodies highly specific for human GSNOR with minimal cross reactivity to other ADH proteins. We verified the presence of GSNOR in ≥85% of specimens examined, and extensive analysis of these samples demonstrated no difference in GSNOR protein expression between cancerous and normal lung tissues. Additionally, GSNOR and other ADH mRNA levels were evaluated quantitatively in lung cancer cDNA arrays by qPCR. Consistent with our immunohistochemical findings, GSNOR mRNA levels were not changed in lung cancer tissues, however the expression levels of other ADH genes were decreased. ADH IB mRNA levels were reduced (>10-fold) in 65% of the lung cancer cDNA specimens. We conclude that the previously reported results showed an incorrect association of GSNOR and human lung cancer risk, and a decrease in ADH IB, rather than GSNOR, correlates with human lung cancer.

## Introduction

S-nitrosoglutathione (GSNO) is an endogenous nitric oxide donor that serves as a depot for nitric oxide (NO) in the body and plays an integral role in communicating NO mediated signaling functions. Decreased levels of GSNO have been correlated to a variety of diseases and its restoration has been proposed as a therapeutic approach to cystic fibrosis [1] and asthma [2], [3]. The oxidoreductase, S-nitrosoglutathione reductase (GSNOR), is the primary enzyme involved in the catabolism of intracellular GSNO, and its pharmacologic inhibition provides a therapeutic mechanism for preserving intracellular GSNO levels. High potency GSNOR inhibitors are currently under clinical development [4]–[7].

GSNOR, also known as the alcohol dehydrogenase class III enzyme (ADH III) and formaldehyde dehydrogenase, is evolutionarily the oldest member of the ADH protein family, and all other ADHs are thought to derive from GSNOR by gene duplication [8], [9]. In humans, the ADH isozymes are homologous and show up to 60% amino acid sequence identity. They are also highly conserved between species. This high amino acid sequence identity creates challenges in developing specific antibodies to GSNOR. Polyclonal antibodies have been used in several publications [3], [10]–[13], and the only commercially available antibodies for GSNOR are polyclonal. For example, Marozkina and colleagues used commercially available polyclonal antibodies against human GSNOR to suggest that decreased GSNOR activity from a therapeutic GSNOR inhibitor could leave the lung vulnerable to oncogenic effects from nitrosative stress [12]. We demonstrate that these polyclonal antibodies do not have sufficient specificity to conclude that the signal observed was due to GSNOR rather than other ADH isozymes. We have developed several highly specific monoclonal antibodies against human GSNOR and used these antibodies to screen arrays of different cancerous and normal lung tissue samples. In addition to immunohistochemistry, we quantitatively measured mRNA levels of GSNOR and other ADH isozymes. We demonstrate that the previously reported signal observed by Marozkina et al. was more likely ADH IB and not GSNOR. Monoclonal antibodies to GSNOR provide a more appropriate tool for characterizing GSNOR protein expression.

## Materials and Methods

### Antibodies and purified proteins

ADH IA protein was purchased from Abnova (Taipei City, Taiwan, # H00000124-Q01), but some degradation was noted in this preparation. Other proteins used in this study were prepared for us by Emerald BioStructures (Bainbridge Island, WA) as described previously [14]. Briefly, GSNOR, ADH IB, ADH II, and ADH IV were expressed with an N-terminal 6×-histidine affinity tag and a Smt-fusion sequence which was removed by Ulp-1 cleavage after Ni affinity chromatography to produce the full-length recombinant proteins. The approximate molecular weights for the ADH proteins before and after removal of the His-Smt tag are: ADH IB (51 kDa, 40 kDa), ADH II (51 kDa, 40 kDa), and ADH IV (53 kDa, 41 kDa). The His-Smt-GSNOR fusion protein is approximately 50 kDa. The GSNOR protein preparations used for antibody generation were confirmed by Emerald BioStructures to be full length by mass spectrometry [4] with a molecular weight of 39.6 kDa. We and others [15] have shown that GSNOR, whether purified or from human cell lysates, consistently migrates slightly faster than its predicted molecular weight on SDS-PAGE. A polyclonal antibody was generated from serum of rats immunized with purified, recombinant, full length human GSNOR protein at Biomodels (Watertown, MA) for N30 Pharmaceuticals. Three distinct monoclonal antibodies to human GSNOR (N30-C3 rat anti-GSNOR, N30-F6 mouse anti-GSNOR, and N30-G11 mouse anti-GSNOR) were generated by immunization of mice or rats with purified, recombinant, full length human GSNOR protein at ProMab Biotechnologies (Richmond, CA) for N30 Pharmaceuticals. Serum titer was evaluated by ELISA prior to selection for hybridization. Hybridoma fusion with myeloma cells (ProMab Biotechnologies; Richmond, CA) was performed with the splenocytes from the mouse or rat with the best titer. Supernatants from the hybridoma wells were screened by ELISA for positive reactivity for GSNOR. Supernatants were also screened by ELISA for negative reactivity for ADH IB, ADH II and ADH IV. Supernatant from 10 clones was then screened at N30 Pharmaceuticals by western blot for specificity for the GSNOR isozyme and minimal non-specific protein binding. Two of the 10 clones for both the mouse and the rat were selected and then subcloned at ProMab by limiting dilution. For the mouse monoclonal antibodies, a supply of antibody was prepared by ascites production in 10 mice, and the antibody was harvested and purified by IgG chromatography. For the rat monoclonal antibody, antibody was produced in vitro by cell culture and IgG purified. Rabbit polyclonal anti-GSNOR was purchased from Proteintech (Chicago, IL; #11051-1-AP).

### GSNOR immunoblotting

Purified ADH proteins (22.5 ng/well) were analyzed by SDS-PAGE and immunoblotting using the polyclonal antibody (Proteintech; 1∶50 dilution to final concentration of 1.4 µg/mL) or monoclonal antibodies (rat anti-GSNOR N30-C3, 2.4 µg/mL; mouse anti-GSNOR N30-F6 and N30-G11 10 ng/mL). HRP conjugated secondary antibodies (Santa Cruz Biotechnology, Santa Cruz, CA) were used for detection.

### GSNOR immunohistochemistry

Immunohistochemical analysis for GSNOR was performed using formalin-fixed, paraffin-embedded human lung tissue microarrays (Folio Bioscience, Columbus, OH; #ARY-HH0178, #ARY-HH0176). Slides were deparaffinized in xylene and rehydrated to water through a series of alcohol gradients. The slides were pretreated in a 95°C citrate pH 6.1 antigenic retrieval solution (Dako, Carpinteria, CA). After allowing the slides to cool to room temperature, a 3.0% hydrogen peroxide solution (EMD Chemicals, Gibbstown, NJ) was added to quench endogenous peroxidase activity. Slides were preincubated with Serum Free Protein Block (Dako), followed by incubation with a GSNOR antibody or the appropriate non-specific immunoglobulin control: rat IgG control (AbD Serotec, Raleigh, NC), mouse IgG2B (Dako), or rabbit Ig fraction (Dako). Slides labeled with the N30-C3 antibody were then incubated with rabbit anti rat immunoglobulin (Dako), and all slides were then labeled with a goat anti rabbit or anti mouse polymer (Dako). DAB+ (Dako) was used for detection, and slides were counter stained with hematoxylin (Dako).

### Analysis of mRNA levels in lung tissue

cDNA arrays made from 23 matched pairs of normal and cancerous lung tissue were purchased from Origene (Rockville, MD; #HLRT504, lot #0411). The pairs included lung cancer tissue covering Stage IA (n = 4), IB (n = 4), IIA (n = 2), IIB (n = 8), IIIA (n = 3), and IIIB (n = 2) each paired with adjacent normal tissue. One Stage IV tissue sample was not paired with a normal sample, and was not included in the analysis. Gene expression of GSNOR (ADH III; gene name *ADH5*), ADH IB (gene *ADHIB*), ADH II (gene *ADH4*), ADH IV (gene *ADH7*), and beta-actin was quantified by qPCR using TaqMan primer probe sets (Applied Biosystems, Carlsbad, CA): Human B-Actin (Hs00357333_g1), Human GSNOR (Hs00605185_m1), Human ADH IB (Hs00605175_m1), Human ADH II (Hs00923466_m1), and Human ADH IV (Hs00609447_m1). Quantitative PCR (qPCR) was performed with an ABI 7300 RT PCR instrument (Applied Biosystems) using Perfecta qPCR fastmix (Quanta Biosciences, Gaithersburg, MD) according to the manufacturer's instructions. Samples were subjected to 1 cycle at 95°C for 3 minutes, followed by 40 cycles at 95°C for 15 sec and 60°C for 1 minute. For data analysis, comparative threshold cycle values (C_t_) for beta-actin were used to normalize loading variations and calculate ΔC_t_ values. ΔΔC_t_ values were calculated by subtracting the ΔC_t_ value for the control (normal) tissue from the ΔC_t_ value for the matched tumor tissue. ΔΔC_t_ values were converted to fold differences using the formula 2^−ΔΔCt^.

## Results

### Commercial polyclonal GSNOR antibody cross-reacts with other ADH proteins

**Figure 1:**
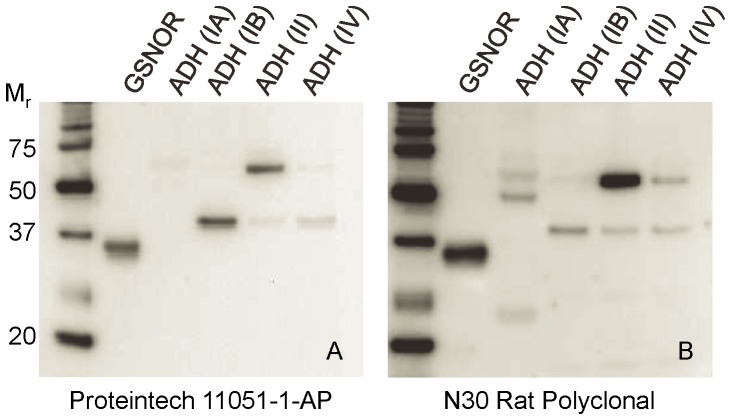
Polyclonal GSNOR antibodies react with other ADHs. 22.5 ng purified recombinant proteins were separated by SDS-PAGE, and immunoblots were performed to determine antibody reactivity to GSNOR, ADH IA, ADH IB, ADH II, and ADH IV. With the exception of ADH IA, the purified recombinant proteins were generated with a fusion protein tag during cloning, and the tag was cleaved off during purification as described in the Materials and Methods. The molecular weights of the fusion proteins are approximately 50–51 kDa, and the final, purified proteins are 39–41 kDa. Human GSNOR consistently migrates faster than the calculated molecular weight. As seen by for the presence of both 50 and 40 kDa bands for ADH II, the majority of the protein in this preparation still contains the fusion protein tag. However, the purified GSNOR protein used for immunization was confirmed to be full length, and free of additional tag sequence as described in the Materials and Methods. A) Commercially available rabbit polyclonal GSNOR antibody (Proteintech #11051-1-AP). B) In-house polyclonal antibody generated by immunization of rats with purified, recombinant, full length human GSNOR protein at Biomodels (Watertown, MA) for N30 Pharmaceuticals.

**Figure 2:**
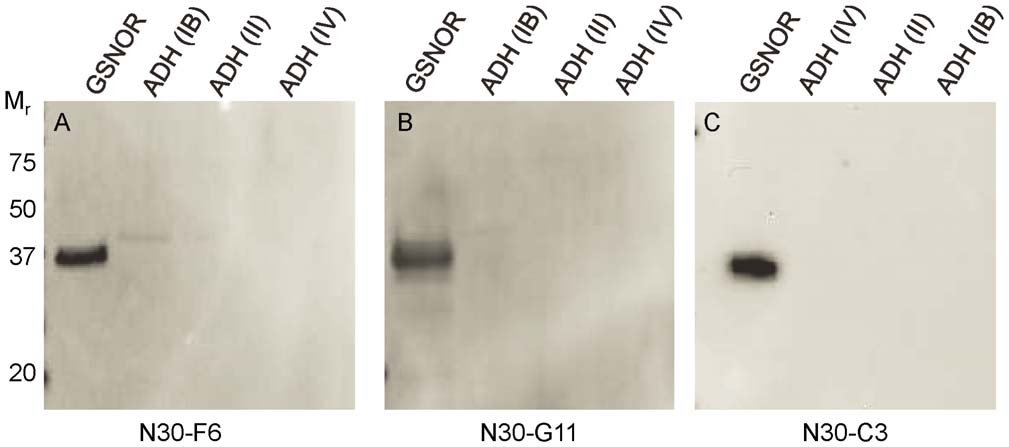
Monoclonal GSNOR antibodies are specific for GSNOR. Purified recombinant proteins were separated by SDS-PAGE, and immunoblots were performed to determine antibody reactivity to GSNOR, ADH IB, ADH II, and ADH IV. Three independent monoclonal antibodies were tested: A) N30-F6 mouse anti-GSNOR; B) N30-G11 mouse anti-GSNOR; C) N30-C3 rat anti-GSNOR.

Recent work examining GSNOR in human lung cancer [12] employed immunohistochemical staining of human lung cancer samples with a commercially available polyclonal GSNOR antibody (see materials and methods). Because the protein sequence of GSNOR is highly conserved with other ADH isozymes, we determined its specificity for GSNOR compared with its specificity toward other ADHs. Cross reactivity was examined by immunoblotting using purified human recombinant GSNOR (ADH III), ADH IA, ADH IB, ADH II, and/or ADH IV. As shown in Figure 1A, a commonly used commercial polyclonal antibody strongly cross-reacts with other ADH proteins, particularly ADH IB, ADH II, and ADH IV. We tested other commercially available polyclonal antibodies and an in-house prepared rat polyclonal GSNOR antibody and found all polyclonal antibodies tested also strongly cross-reacted with ADH proteins (data not shown and Figure 1B). To address this issue, monoclonal antibodies specific for GSNOR were developed by immunization of mice and rats with purified, full length, recombinant human GSNOR as described in the methods. This effort also included a counter-screening step to minimize cross reactivity to other ADH proteins. As shown in Figure 2, three of the new monoclonal antibodies against GSNOR have either greatly reduced or no cross reactivity to other ADH proteins. To our knowledge, this is the first paper to describe antibodies with this level of specificity toward GSNOR.

### Monoclonal GSNOR antibodies detect expression in a wide variety of human lung cancers

**Figure 3:**
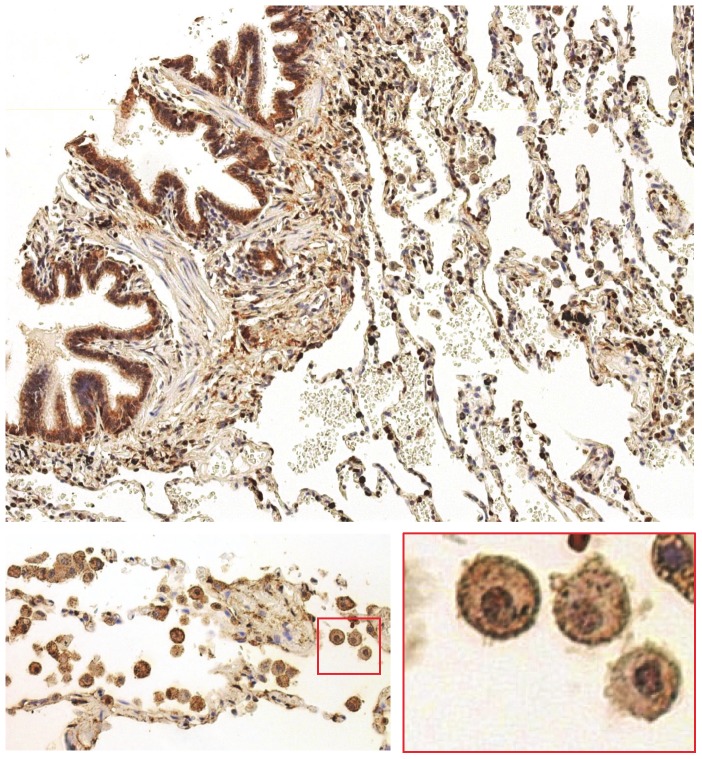
GSNOR is present in normal lung tissue. Normal human lung tissue microarrays were stained with N30-C3 monoclonal GSNOR antibody (1 µg/mL) followed by DAB detection. Bronchial epithelial cells, alveolar macrophages, and type 2 pneumocytes in alveoli are strongly stained.

**Figure 4:**
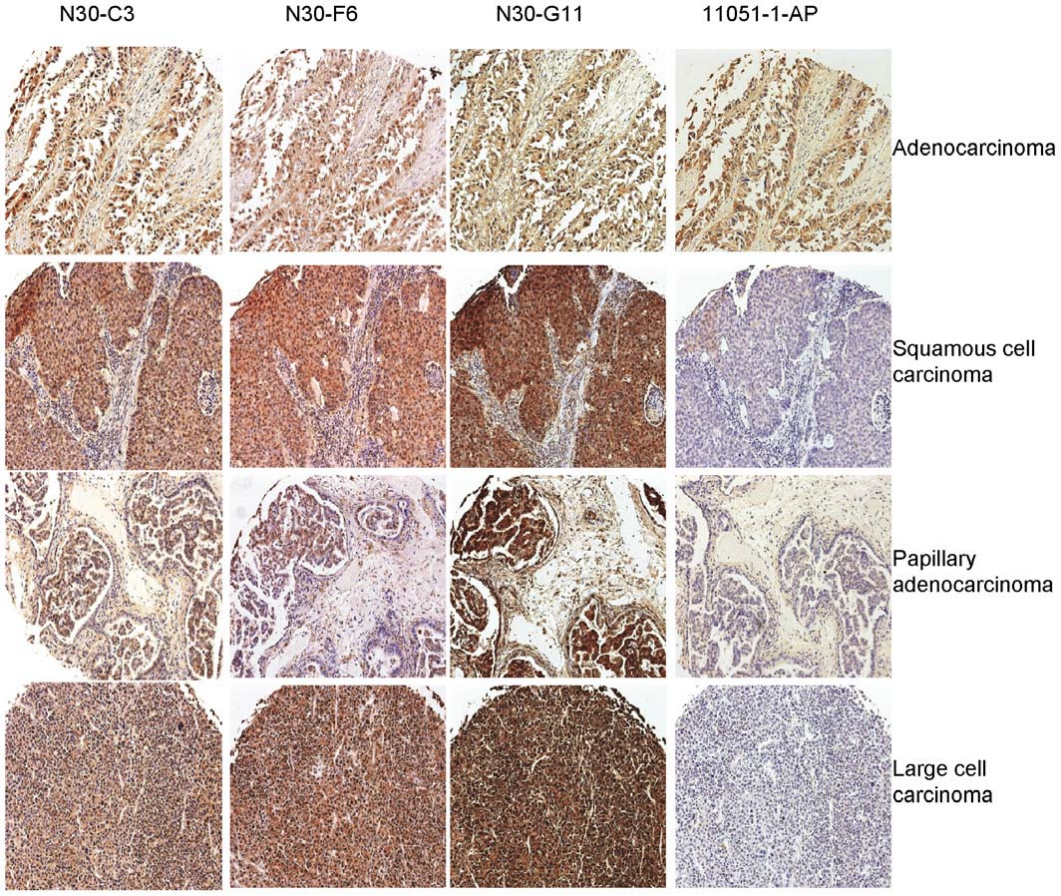
GSNOR is found in human lung cancer tissues stained with monoclonal GSNOR antibodies. Normal human lung cancer tissue microarrays were stained with monoclonal GSNOR antibodies (N30-C3, N30-F6, N30-G11) or a commercially available polyclonal GSNOR antibody (11051-1-AP) followed by DAB detection. Various human lung cancer tissues are strongly stained by all three GSNOR monoclonal antibodies, but staining is less prominent when the non-specific polyclonal GSNOR antibody is used.

We examined GSNOR protein levels in various lung cancer tissues by immunohistochemistry using the above described three GSNOR-specific monoclonal antibodies and compared the tissue staining pattern to the commercially available polyclonal antibody used in a previous publication [12]. We also compared the antibody signal in normal lung tissue. Using the four antibodies, we stained four identical human lung cancer tissue microarrays each containing human tissue cores from over 100 cases of lung cancer at various stages of progression. GSNOR was strongly detected in normal human lung tissue including in bronchial epithelial cells, alveolar macrophages, and type 2 pneumocytes in alveoli (Figure 3). GSNOR was also detected in a wide variety of lung cancer tissues including adenocarcinoma, squamous cell carcinoma, papillary adenocarcinoma, and large cell carcinoma when tissues were stained with the monoclonal GSNOR antibodies (Figure 4, N30-C3, N30-F6, N30-G11). Staining in lung cancer tissues was fainter when the polyclonal antibody was used (Figure 4, 11051-1-AP). Tissue sections were scored by three reviewers for GSNOR staining for each antibody (Table 1). Using the three specific monoclonal antibodies, GSNOR protein levels were not different in lung cancer specimens compared to normal tissue; whereas the polyclonal antibody with demonstrated ADH cross reactivity suggested that GSNOR expression was higher in normal lung tissue.

**Table 1:** Quantitation of GSNOR positive lung cancer tissue sections.

Antibody	% tumors positive for GSNOR	% normal lung positive for GSNOR
N30-C3	85	83
N30-F6	95	83
N30-G11	99	92
11051-1-AP	28	83

Four identical sets of 120 lung tissue cores (12 normal, 108 cancer) were stained with the GSNOR antibodies indicated below. The stained tissue cores were graded as positive or negative for GSNOR expression by three reviewers blinded to antibody used (SCM, GJR, JPR). The percent of tumor and normal lung cores graded as positive for GSNOR expression are shown below.

### GSNOR mRNA levels are not decreased in human lung cancer tissue versus adjacent normal tissue

**Figure 5:**
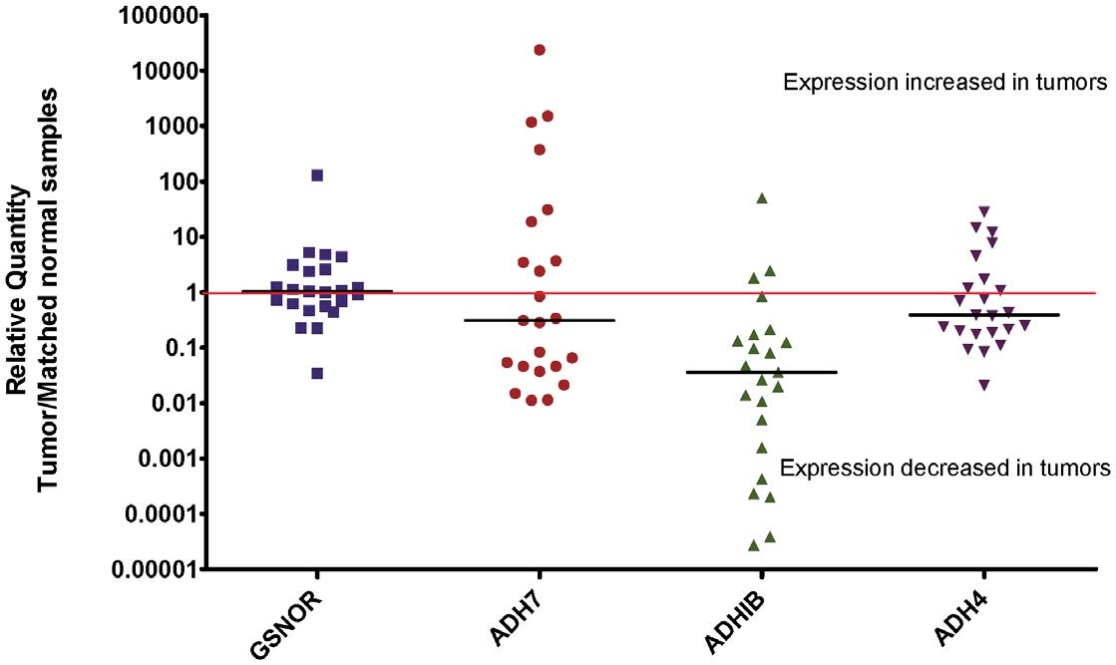
GSNOR and ADH gene expression in lung cancer cDNA arrays. GSNOR (gene name *ADH5*), ADH IB (*ADHIB*), ADH II (*ADH4*), and ADH IV (*ADH7*) mRNA levels were evaluated by qPCR in human lung cancer cDNA arrays (Origene, #HLRT504, lot #0411, Rockville, MD) and normalized to β-actin levels. Arrays contained 23 matched pairs of normal and cancerous lung tissue. Tumor expression relative to the matched normal sample was calculated using the ΔΔCt method. Relative quantities <1 represent decreased expression in the tumor sample. Lung cancer specimens included Stage IA (n = 4), IB (n = 4), IIA (n = 2), IIB (n = 8), IIIA (n = 3), and IIIB (n = 2) with each sample paired with adjacent normal tissue. No correlation between GSNOR mRNA levels and lung cancer was observed, while expression of ADH IB was strongly reduced in lung cancer samples.

Due to the potential for subjectivity in grading tissue staining, GSNOR and ADH mRNA levels were evaluated quantitatively in human lung cancer cDNA arrays. Commercially available cDNA arrays (see materials and methods) were used that contained 23 matched pairs of normal and cancerous lung tissue. The pairs included Stage IA (n = 4), IB (n = 4), IIA (n = 2), IIB (n = 8), IIIA (n = 3), and IIIB (n = 2) lung cancer tissues each paired with adjacent normal tissue. GSNOR mRNA levels were quantitated using qPCR, and relative quantities of GSNOR in tumor versus adjacent normal tissue were calculated as described in the supplementary methods. No correlation was observed between GSNOR mRNA expression and lung cancer. In this analysis, 11 of 23 tumor samples had relative tumor/normal expression ratios <1 and 12 of 23 samples had expression ratios >1. Furthermore, evidence of dramatically decreased GSNOR expression was rare in the tested lung cancer samples; only 3 of the tumor samples were found with GSNOR expression decreased by more than 3-fold (ratio <0.33; Figure 5). Conversely, when expression of ADH IB, ADH II, and ADH IV was analyzed, more of the samples demonstrated larger expression decreases in the tumor samples compared to GSNOR. Moreover, we observed substantial decreases in ADH IB expression in lung cancer tissues, with 12 of 23 pairs having ≥10-fold decrease in ADH IB expression in the lung cancer samples (relative tumor/normal expression ratio ≤0.1). Only 3 of 23 pairs had increased expression of ADH IB in the lung cancer samples compared to the adjacent normal tissue (Figure 5).

## Discussion

In the lung, endogenous S-nitrosothiols including GSNO mediate NO-based signaling, inflammatory responses, and smooth muscle function. Inhibition of GSNOR represents a novel approach to treating respiratory disease via preserving endogenous GSNO, and a novel GSNOR inhibitor is currently in clinical development for the treatment of asthma and cystic fibrosis [4].

In this study, we demonstrate no consistent differences in the expression of GSNOR when comparing lung cancer tissue to normal lung tissue. These findings contradict previously published work by Marozkina and colleagues [12] who reported that GSNOR expression was decreased in lung cancer tissues. In their analysis, the authors observed that 69% of human lung cancer biopsies demonstrated significantly reduced GSNOR levels. In their assessment, GSNOR levels were significantly lower in late stage tumors compared to early stage tumors, and they concluded that reduced GSNOR activity is correlated with later stages of tumor maintenance. It is likely that a primary discrepancy between our report and previous reports stems from the lack of specificity of the polyclonal antibodies used in their analysis. We demonstrated that the polyclonal antibody strongly cross reacts with other, closely related proteins, including ADHIB, ADH II, and ADH IV. In fact, we have found that every polyclonal antibody we have tested suffers from this same lack of specificity, and conclusions about GSNOR expression and localization drawn from experiments using polyclonal antibodies have great potential to be misinterpreted. The lack of specificity in polyclonal antibodies is not surprising given the high sequence identity between GSNOR and other ADH isozymes. When developing a monoclonal antibody to GSNOR, we found it necessary to counter-screen the clones against several other purified ADH proteins to obtain the required specificity. Using GSNOR specific monoclonal antibodies, we found no difference in the staining of cancerous versus normal lung tissue.

Additionally, we explored the expression of not only GSNOR but also other ADHs in lung cancer tissue using more definitive qPCR methods. No change in GSNOR mRNA expression between normal and cancerous lung tissue was observed. Conversely, when expression of ADH IB, ADH II, and ADH IV were analyzed, we saw more and larger expression decreases in the tumor samples. Moreover, we observed substantial decreases in ADH IB expression in lung cancer tissues with 12 of 23 pairs having ≥10-fold decrease in ADH IB expression in the lung cancer samples (relative tumor/normal expression ratio ≤0.1).

That ADH IB expression shows some relationship to lung cancer is not surprising. ADH IB is integral in a number of pathways with known influence in lung cancer including fatty acid, retinol and tyrosine metabolism, as well as in glycolysis and gluconeogenesis [16]. In a meta-analysis of published studies looking at the relationship of ADH IB in lung cancer, ADH IB expression was decreased in lung cancer in an overwhelming number of samples and analysis [16] and clearly polymorphisms of ADH IB have shown correlations to other types of cancer including esophageal [17]. Unlike ADH IB where there has been substantial investigation in its relation to cancer, GSNOR expression levels have recently been suggested to correlate with hepatocellular carcinoma [13]. These studies in part relied on GSNOR knock-out mice that have substantial phenotypic differences when compared to wild type controls, and recent studies from our lab have shown this any purported relationship between genetic deletion of GSNOR and hepatocellular carcinoma is not seen in a haplosufficient model (GRJ and D. Colagiovanni, manuscript in preparation, N30 Pharmaceuticals).

In summary, it appears that a decrease in ADH IB, rather than GSNOR, correlates with human lung cancer, and the previously reported results showed an incorrect association of GSNOR and human lung cancer risk. In further support of this conclusion, published studies of mice with homozygous genetic deletion of GSNOR have found no increase in murine lung cancer in both untreated GSNOR^−/−^ mice nor GSNOR^−/−^ mice treated with the carcinogen diethylnitrosamine [13]. Thus GSNOR expression and inhibition do not appear to be associated with risk of human lung cancer.
